# Microbial Changes in Subgingival Plaque and Polymicrobial Intracellular Flora in Buccal Cells after Fixed Orthodontic Appliance Therapy: A Preliminary Study

**DOI:** 10.1155/2013/679312

**Published:** 2013-10-02

**Authors:** Caterina Montaldo, Matteo Erriu, Francesca Maria Giovanna Pili, Carla Peluffo, Annalisa Nucaro, Germano Orrù, Gloria Denotti

**Affiliations:** ^1^Department of Surgical Sciences, University of Cagliari, 09121 Cagliari, Italy; ^2^Neurogenetic and Neuropharmacology Institute, Italian National Research Council, 09121 Cagliari, Italy

## Abstract

The oral ecosystem is strictly related to a balance maintained by specific niches recognized as sites, where oral bacteria can metabolize avoiding the immune system response. The oral bacteria species that colonize the ecological niches vary during fixed orthodontic treatment, with a prevalence of periodontal bacterial species. Qualitative analysis of five periodontal pathogens was used to investigate the microbial colonization rate in the crevice and buccal epithelial cells. The presence of inadequate oral hygiene was considered as a modulation variable for microbial colonization. Statistical analysis was performed by Fisher's exact test, ANOVA, and Pearson correlation. A *P* value lower than 0.05 was assumed as statistically significant. *Tannerella forsythia* was the only periodontal pathogen detected with a statistically admissible frequency. The positivity for *Tannerella forsythia* was correlated to sampling time and oral hygiene motivation. In buccal epithelial cells, both factors contributed to microbial decrease (*P* < 0.05), whereas, in crevice, oral hygiene motivation promoted a decrease in the microbial colonization rate (*P* < 0.05). According to microbiological findings, it is possible to identify how correct motivation for oral hygiene is more than enough to modulate or to avoid an upset of the oral ecosystem balance in early stages of orthodontic treatment.

## 1. Introduction

The oral cavity represents a complex and highly organized ecosystem, relied on a delicate balance between the environment and oral microorganisms. In fact, the existence of specific ecological niches, where bacteria can grow and metabolize, regulates the maintenance of this balance and prevents environmental changes [1–3]. However, in cases where the oral ecosystem is altered, an uncontrolled colonization of bacteria can promote the consequent generation of pathological conditions [2, 3]. The tonsils, tongue crypts, and periodontal pockets (PP) are considered as the three most important niches in the oral cavity. In recent years, buccal epithelial cells (BEC) have received consideration as further ecological niches. In 2001, Rudney et al. demonstrated that periodontal bacteria can metabolize in other sites than the gingival crevice. Moreover, as a result of bacterial species colonization, they suggested that BEC become sites where pathogens can metabolize completely undisturbed and then, from which, they can move on to colonize other areas [4]. In particular, *Aggregatibacter actinomycetemcomitans* and *Porphyromonas gingivalis* have been identified as being mainly involved in the colonization of mucosal cells. In 2006, the same group showed how *Tannerella forsythia* can also invade the BEC and highlighted the strong influence that fixed orthodontic treatment can have on the microbial invasion of the ecological niches. Additionally, according to the reported data, the advancement of orthodontic therapy increased the bacterial count in both the gingival crevice and the BEC [5].

Orthodontic treatment can greatly affect the properties of the oral environment and, subsequently, alter the equilibrium of the microorganism ecosystem, increasing the potential for pathogenicity within the microbial ecosystem. This influence is linked to multiple factors related to the mechanical and structural characteristics of the orthodontic devices, to the consequences of orthodontic movements, and to oral hygiene practices [6–9]. In fact, the effects of stress on the periodontal tissues, the difficulties in maintaining an adequate standard of oral hygiene in the presence of brackets and bands, and the frequent abrasions and ulcerations of the soft tissues, which increase the capacity of periodontal bacteria to penetrate epithelial cells, could probably be mitigated by the strict control and conditioning of specific domiciliary oral hygiene protocols [10, 11]. Although a strict control of oral hygiene would contrast the negative influence of the device [2, 9], there is no work in the literature that describes the effect of oral hygiene motivation on BEC during fixed orthodontic treatment [6, 8, 10–12]. 

The aim of this preliminary work is to investigate the role of oral hygiene instruction in the modulation and control of bacterial colonization of the gingival crevice and BEC during fixed orthodontic treatment.

## 2. Materials and Methods

### 2.1. Patient Selection

A sample of 19 patients (12 female, 7 male) aged from 10 to 22 years (mean age, 13.3) was recruited by the following criteria: 22 years old or under, no history of periodontal disease and absence of periodontal pockets, medically healthy, presence of maxillary second premolars, and treatment planned with maxillary and mandibular fixed orthodontic appliances. Patients who had undergone antibiotic therapy in the 6 months preceding the periodontal examination were not included in the study. Furthermore, all participants were informed about the research and its purpose, and, gave their informed consent in accordance with the ethical standards of the Helsinki Declaration.

### 2.2. Clinical Evaluation, Motivation, and Samples Collection

All clinical examinations were carried out monthly for a total of 4 visits. Assessment of periodontal parameters (plaque index, bleeding on probing, pocket depth, and attachment loss) and microbial sampling were regularly recorded at each time point—before the placement of the fixed appliance (*T*0), after 1 month (*T*1), after 2 months (*T*2), and after 3 months (*T*3). 

In addition, at the time of the first evaluation, immediately prior to the placement of the device, the clinician provided the patients with information specifically regarding oral health maintenance during an orthodontic treatment. After one month, the patients did not receive any additional recommendations for routine mouth care. After two months, in order to promote behavioural change, specific guidelines related to domiciliary oral hygiene practices were addressed and renewed. Lastly, three months after the start of orthodontic therapy, the clinician reexamined the efficacy of the previously proposed preventive programme and its reflections on oral health maintenance.

In order to confirm the presence of periodontal bacteria within epithelial cells from buccal mucosa, brush cytology samples from both cheeks were collected in the area next to the second upper premolars. The brushes were then inserted into tubes containing 1 mL of DNAsi-RNAsi free water. The suspension was stored at −20°C and successively used for DNA extraction.

In the second instance, the subgingival plaque samples were taken from the PP of the two upper second premolars (1.5 and 2.5). First of all, the sample area was isolated using sterile cotton rolls and air-dried to avoid saliva contamination, and a sterile paper point ISO 45 (Roeko Dental, Langenau, Germany) was inserted into the pocket and held in place for 30 seconds. The paper point was then removed and placed into a vial containing 50 *μ*L of dimethyl sulfoxide (DMSO) and stored at −20°C to be successively used for molecular analysis [13, 14]. 

The occurrence of *Porphyromonas gingivalis (Pg)*, *Prevotella intermedia (Pi)*, *Aggregatibacter actinomycetemcomitans (Aa)*, *Tannerella forsythia (Tf),* and *Treponema denticola (Td)* was therefore investigated by polymerase chain reaction (PCR).

### 2.3. Assessment and Instruction of Oral Hygiene

At the first visit, which preceded at least a month the insertion of the orthodontic appliance (*T*0), the oral health status of patients was evaluated. All patients included in the study were subjected to a ultrasonic instrumentation and were provided with the techniques of maintaining oral health in the absence of orthodontic appliance. At this stage, the instructions were based on teaching the technique of brushing and using dental floss and interdental toothbrushes. A low abrasive toothpaste, not containing triclosan, was also been prescribed. The use of mouthwashes or other products with antibacterial properties were forbidden to patients.

At *T*0, after an assessment of the state of oral health, new oral hygiene instructions were given to the patients. These were primarily related to the brushing technique in the presence of a fixed orthodontic appliance and the type of toothbrush to use. Both the interdental toothbrush and a dental floss with rigid ends have been prescribed for the maintenance of oral health in the areas between the teeth. Even at this stage the use of mouthwashes or any other product with antibacterial ability have been prohibited.

### 2.4. DNA Extraction

Genomic DNA from clinical samples was obtained by the CTAB modified method. 400 *μ*L of each sample was added to 70 *μ*L of 10% Sodium Dodecyl Sulphate (SDS) and 5 *μ*L of Proteinase K at 10 mg/mL concentration. After vigorous vortex mixing, the mixture was incubated for 10 minutes at 65°C. Next, 100 *μ*L of NaCl [5 M] and 100 *μ*L of CTAB/NaCl (0.274 M CTAB, Hexadecyl Trimethylammonium Bromide and 0.877 M NaCl) were added into the tube and incubated at 65°C for 10 minutes. 750 *μ*L of SEVAG (Chloroform: Isoamyl Alcohol 24 : 1) was added. After centrifugation for 5 min (at 12000 rpm), 0.6 volumes of Isopropanol were added to the supernatant. After 30 min at −20°C, and after being centrifuged for 30 min at 12.000 rpm, the pellet was dried at room temperature for 20 min and suspended in 40 *μ*L of DNAsi-RNAsi free water. 5 *μ*L of this was used as DNA suspension for conventional PCR.

### 2.5. PCR Amplification

Detection of bacterial DNA was performed with the amplification of the bacterial 16S rDNA sequence.

For the PCR reaction, we used selection primers (Table 1) specific for bacterial DNA, even in the presence of nucleic acid from other sources. 

PCR was carried out using an Eppendorf thermocycler, and the reaction was performed in a final volume of 25 *μ*L containing 6.0 mM MgCl_2_, 0.6 nM of each primer pair, SmarTaq Mastermix (Fisher Molecular Biology, Trevose, USA). PCR conditions were initial denaturation at 95°C for 3 minutes; 40 cycles of 95°C for 1 minute, 50°C for 1 minute, and final extension at 68°C for 3 minutes and 40 seconds. The annealing temperature was 49°C for *Aa*, 50°C for *Tf *and *Pg, *and 55°C for *Pi*. A negative control reaction without template DNA was included in each PCR. PCR products were analysed by electrophoresis in 2% agarose gel (Fisher Molecular Biology, Trevose, USA) performed at 150 V in 10X TAE buffer. The gel was stained with Loading Buffer and photographed under 300 nm ultraviolet light. A 100 bp DNA ladder was used as the molecular weight marker [15].

### 2.6. Statistical Analysis

Analyses were performed using Minitab 16.1.1 software. Results were analyzed by using, on one hand, Pearson correlation, which assesses whether two continuous variables are linearly related, and, on the other, and analysis of the variance (ANOVA); *P* values <0.05 were considered statistically significant. 

## 3. Results

On the base of the molecular findings, *Tannerella forsythia* was the only pathogen identified with a sufficient frequency to apply the statistical methods (*f*: 18%, Table 2). The presence of other pathogens appears to be sporadic and not statistically significant (*f*: 1.7%, *P* value > 0.05).

**Figure 1:**
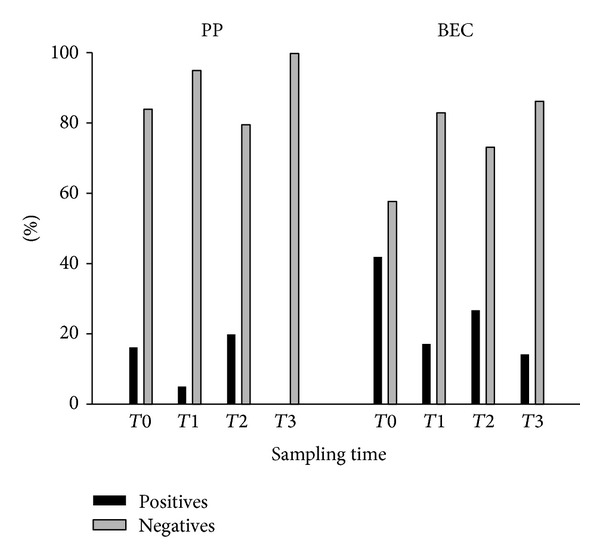
Distribution of positivity divided by site and sampling time.

**Figure 2:**
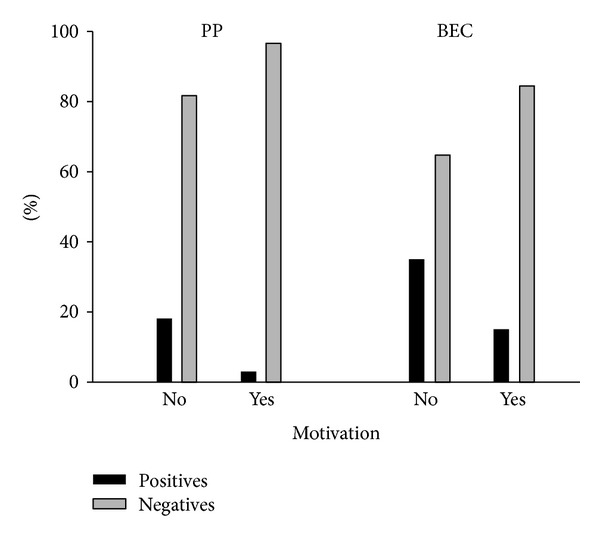
Distribution of positivity divided by site and motivation for the oral hygiene.

Firstly, ANOVA was performed after the unstack of variable microbial positivity for the factor sampling site. Consequently, the presence or absence of *Tf* was compared with the different sampling times, as well as the presence or absence of motivation for oral hygiene. According to ANOVA, the results were always significant (*P* value < 0.05) (Figures 1 and 2, Table 3).

In a second instance, the same criteria were adopted to perform Pearson correlation. From the data obtained, bacterium positivity was influenced by the presence of a motivation for oral hygiene for each site, whereas the sampling time was only correlated with positivity in the BEC (Table 4).

## 4. Discussion

The microbiological imbalance derived from orthodontic therapy can be associated with several factors, such as the application of orthodontic forces and, consequently, stress on the connective ligament and periodontal attachment and the presence of brackets and bands which increase plaque buildup and possible injury to the soft tissues [7]. In recent years, several studies have demonstrated that, during fixed orthodontic treatment, two ecological niches are particularly exposed to microbiological changes, namely, PP and BEC [9]. In addition, progression of the therapy may enhance the proliferation of bacterial species. Other works have suggested that the microbial colonization of the PP, in the course of orthodontic treatment, could be modulated, controlled, and also diminished through a tight control on the patient's domiciliary oral hygiene [7, 12]. In this work, it is possible to observe how the modification of the oral ecosystem and, in particular, the increase or decrease of *Tf* are strictly linked to both the fixed orthodontic treatment and also the presence of oral hygiene motivation. This correlation is reported in Figure 1, where positivity for *Tf* increased in correspondence to the phases not preceded by a conditioning of the patient's oral hygiene behaviour (*T*0 and *T*2). At these stages, the positive sites show an upward trend, confirming that fixed orthodontic treatment entails an increasing presence of bacteria in periodontal sites [7, 8] and in the BEC [9]. 

The increase of positive sites related to the effect of orthodontic therapy is counteracted by the presence of a motivation for oral hygiene. Such an influence is quantitatively more evident in PP, where the number of positive sites at *T*3 drops to 0. At the same time however, a contradiction emerges in the results related to microbial presence in the PP. In fact, ANOVA demonstrated how the variance of the presence of the bacterium is statistically influenced by the duration of the therapy, as well as by motivation. On the other hand, it is not possible to find a statistical correlation between the decrease in *Tf* and the time sampling. This contradiction can be explained by observing the trend of the microbiological results related to PP. It is strongly oscillating and this is explicitly correlated with the presence or absence of motivation for oral hygiene. When it is absent, the effect of orthodontic treatment becomes evident with a consequent increase in positive sites. From these observations, it can be assumed that PP are highly unstable ecological niches. 

On the contrary, the analysis performed on BEC shows a much more regular trend. ANOVA highlighted a behaviour similar to that of PP, but, in this case, the descending trend of the microbial positivity was statistically correlated with sample time. This result could be explained by the influence of oral hygiene on the microbial colonization of BEC. Probably, the mechanical abrasion produced by fixed orthodontic appliances, which is described in the literature as a favoring factor for BEC invasion, also increases the penetration of antiseptic products with a consequent reduction in the bacterial load. These observations could make it possible to suspect how BEC seem to be ecological niches strongly influenced by the patient's oral hygiene.

## 5. Conclusions

In conclusion, this preliminary study shows that bacterial colonization can be easily modulated and controlled by a good motivation for oral hygiene during fixed orthodontic treatment. These results will need to be verified by further studies on more patients and different age ranges in the early stages of orthodontic treatment.

## Figures and Tables

**Table 1:** Primers used and strains detected by conventional PCR.

Bacteria	Primers	Sequence
*Aa 652 *	OG 155	F: 5′-CATTCTCGGCGAAAAAACTA-3′
	OG 156	R: 5′-CCCATAACCAAGCCACATAC-3′
*Aa JP2 *	OG 155	F: 5′-CATTCTCGGCGAAAAAACTA-3′
	OG 156	R: 5′-CCCATAACCAAGCCACATAC-3′
*Aa Y4 *	OG 155	F: 5′-CATTCTCGGCGAAAAAACTA-3′
	OG 156	R: 5′-CCCATAACCAAGCCACATAC-3′
*Pg *	OG 94	F: 5′-GAATCAAATACTTCAGCCGTCT-3′
	OG 95	R: 5′-TTGCAGTTCGTATCGGATCT-3′
*Pi *	OG 53	F: 5′-CGTATCCAACCTTCCCTCC-3′
	OG 54	R: 5′-ATTAGCCGGTCCTTATTCGAAG-3′
*Td *	OG 348	F: 5′-AGAGAAAGGGTAATTTGAAG-3′
	OG 349	R: 5′-TATTATTGTCCCTTCTTTCTT-3′
*Tf *	OG 45	F: 5′-GTCGGACTAATACCTCATAAAACA-3′
	OG 46	R: 5′-TCGCCCATTGACCAATATA-3′

*AA 652: Aggregatibacter actinomycetemcomitans tp 652*.

*Aa JP2: Aggregatibacter actinomycetemcomitans tp Jp2*.

*Aa Y4: Aggregatibacter actinomycetemcomitans tp Y4*.

*Pg: Porphyromonas gingivalis*.

*Pi: Prevotella intermedia*.

*Td: Treponema denticola*.

*Tf: Tannerella forsythia*.

**Table 2:** Distribution of positivity by site and sampling time.

Sampling time	Motivation	Site	Positives	Negatives	Fisher (*P* value)
0	No	PP	6	30	0.001
1	Yes		2	34	
2	No		7	29	
3	Yes		0	36	
0	No	BEC	15	21	
1	Yes		6	30	
2	No		10	26	
3	Yes		5	31	

**Table 3:** Evaluation of positivity variance in relation to sampling time and motivation (ANOVA).

Variable	Factor	*P* value
BEC	Sampling time	0.026
PP		0.020
All		0.001
BEC	Motivation	0.003
PP		0.007
All		<0.0005

**Table 4:** Evaluation of correlations.

		Sampling time	Motivation	PD
PP	Pearson	−0.132	−0.250	
	*P* value	0.114	**0.003**	
BEC	Pearson	−0.186	−0.225	0.066
	*P* value	**0.025**	**0.007**	0.435
